# Linking tissues to phenotypes using gene expression profiles

**DOI:** 10.1093/database/bau017

**Published:** 2014-03-13

**Authors:** Anika Oellrich, Sanger Mouse Genetics Project, Damian Smedley

**Affiliations:** ^1^Mouse Informatics Group, Wellcome Trust Sanger Institute, Wellcome Trust Genome Campus, Hinxton, Cambridge, CB10 1SA, UK; ^2^ and Mouse Genetics Project, Wellcome Trust Sanger Institute, Wellcome Trust Genome Campus, Hinxton, Cambridge, CB10 1SA, UK

## Abstract

Despite great biological and computational efforts to determine the genetic causes underlying human heritable diseases, approximately half (3500) of these diseases are still without an identified genetic cause. Model organism studies allow the targeted modification of the genome and can help with the identification of genetic causes for human diseases. Targeted modifications have led to a vast amount of model organism data. However, these data are scattered across different databases, preventing an integrated view and missing out on contextual information. Once we are able to combine all the existing resources, will we be able to fully understand the causes underlying a disease and how species differ. Here, we present an integrated data resource combining tissue expression with phenotypes in mouse lines and bringing us one step closer to consequence chains from a molecular level to a resulting phenotype. Mutations in genes often manifest in phenotypes in the same tissue that the gene is expressed in. However, in other cases, a systems level approach is required to understand how perturbations to gene-networks connecting multiple tissues lead to a phenotype. Automated evaluation of the predicted tissue–phenotype associations reveals that 72–76% of the phenotypes are associated with disruption of genes expressed in the affected tissue. However, 55–64% of the individual phenotype-tissue associations show spatially separated gene expression and phenotype manifestation. For example, we see a correlation between ‘total body fat’ abnormalities and genes expressed in the ‘brain’, which fits recent discoveries linking genes expressed in the hypothalamus to obesity. Finally, we demonstrate that the use of our predicted tissue–phenotype associations can improve the detection of a known disease–gene association when combined with a disease gene candidate prediction tool. For example, *JAK2*, the known gene associated with Familial *Erythrocytosis 1*, rises from the seventh best candidate to the top hit when the associated tissues are taken into consideration. **Database URL:** http://www.sanger.ac.uk/resources/databases/phenodigm/phenotype/list

## Introduction

Despite tremendous efforts in the biological and computational domain to identify disease gene candidates (1–5), almost half of the 7000 defined human genetic disorders are still without an identified cause (6). To find cures and prevention mechanisms for diseases, we need to understand the genetic causes triggering the disease. Studies in model organisms have gained more and more importance in the quest for identifying disease gene candidates because they provide a means to specifically target genes and observe the consequences on an organism scale. Model organism databases, such as the Mouse Genome Informatics Database [MGD; (7)], the International Mouse Phenotyping Consortium [IMPC; (8)] or the Sanger Mouse Genetics Project [Sanger-MGP; (4)], store the results of the biological investigation particular to one species. Determining suitable models for a human disease not only provides insights into the genetic causes of a heritable disease but also enables the identification of potential drug targets (9).

To understand the full impact of a gene as well as a drug, all the causal relationships between gene products resulting in a phenotype need to be understood. Part of understanding the relationships and building reasoning chains from a gene to an organism level is the determination of the location of gene products. Once the products are located, pathways recapitulating the interactions of gene products can be defined (10, 11). For this purpose, the IMPC portal not only includes phenotype information resulting from biological experiments but also includes gene expression data from adult mice (http://www.sanger.ac.uk/htgt/biomart/martview). In the IMPC portal, one mouse line corresponds to the mutation of one single gene of the mouse genome. Each mouse line is further characterized with gene expression data and phenotypes. However, the data are not further analyzed for associations between gene expression patterns and phenotypes. Other resources, e.g. BioGPS (12), only provide gene expression data entirely independent from observed phenotypes.

In an earlier study, Hoehndorf *et al.* (13) annotated 1053 mouse genes with 151 different Neuro Behaviour Ontology concepts and determined enriched concepts for groups of differentially expressed genes. Using this approach, the authors could show that the expression data are sufficient to predict the behavioural differences between the two states.

In our study, we analyzed the gene expression and phenotypic profiles to determine global patterns between expression patterns and phenotypes. Establishing links between tissues and phenotypes will allow us to better understand the connections reaching from a molecular level to the resulting phenotype. Applying the approach will not only show affected anatomical entities that are associated with a phenotype but also help understand what other components are involved in the process resulting in the final phenotype. In some cases, an observed phenotype will simply result from disruption of a gene that is specifically expressed in the affected tissue, e.g. disruption of the cardiac-expressed *MYH7* gene resulting in Familial Hypertrophic Cardiomyopathy (OMIM:192600). In other cases, a systems level approach is required to understand the development of the disease, e.g. obesity is known to at least partially involve perturbations to gene-networks connecting the hypothalamus and metabolic tissues (14).

To assess connections between phenotypes and tissues, we used two different methods and provided all our results through PhenoDigm’s web interface (http://www.sanger.ac.uk/resources/databases/phenodigm/; database: WTSI Mouse Genetics Project (Sanger UK); dataset: MGP Phenotyping; attributes: Adult Expression) (1). We evaluated the obtained results for their biological correctness both manually and automatically.

We have recently demonstrated how mouse phenotype comparisons can be used to prioritize candidate genes resulting from exome analysis of rare diseases (15). The phenotype–tissue associations we provide here can be used to further narrow down candidate lists, i.e. the tissues we associate with the phenotypes can be used to further prioritize the candidates based on their individual expression patterns.

## Methods and Materials

**Figure 1.**
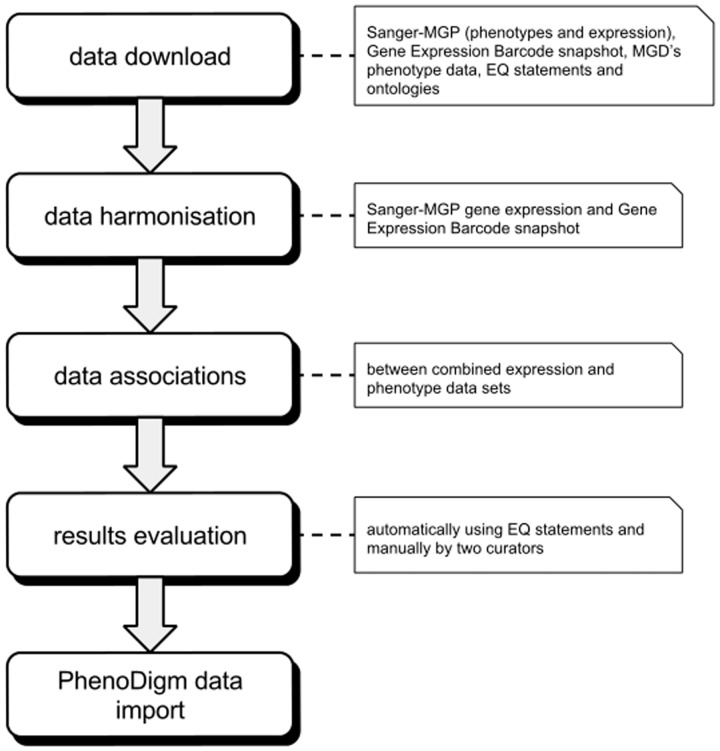
Illustration of the overall work flow of the study. After downloading and formatting all required data, the expression profiles are merged into one data set. The merged data set is then used to calculate the associations between tissues and phenotypes that are then evaluated. After evaluation, the significant associations are loaded into and provided via the PhenoDigm web interface.

Before associating expression and phenotype data, we downloaded and harmonized data sets from several different data repositories. After calculating associations’ scores, we evaluated the obtained associations both automatically and manually. The applied data sets are described in Input data, while the section on Establishing connections between tissues and phenotypes explains the algorithms used for the association of tissues and phenotypes. Implementation focusses on the implementation and Evaluation provides details for the evaluation of the results. The overall work flow of the study is depicted in Figure 1.

### Input data

#### Phenotype annotation data

In our study, we applied the phenotype data that are available from the MGD and Sanger-MGP databases. We downloaded the MGI_GenePheno.rpt report file on 20 July 2013 and accumulated phenotype annotations on a gene level. This data set comprises phenotype annotations for single-gene knockouts only so that a relationship between a gene and the resulting phenotype can be assumed. The Sanger-MGP data was downloaded at the same time from http://www.sanger.ac.uk/htgt/biomart/martview. The applied version of the files comprised 126 522 phenotype annotations for 9447 genes (average 13.391 phenotypes per gene). MGD uses the Mammalian Phenotype Ontology (MP) to represent mouse phenotypes. The data set applied in this study included 7393 unique MP concepts.

#### Expression data

To utilize gene expression in our study, several different data repositories were used to compile a comprehensive amount of tissue data. We used the Sanger Mouse Genome Portal (Sanger-MGP) expression data in combination with the Gene Expression Barcode database (http://barcode.luhs.org/) (16).

Sanger-MGP provides not only expression data but also phenotype annotations on a mouse line level. Each mouse line is characteristic for a particular gene knockout and characteristic phenotypes are determined by applying 20 standard operating procedures (17). For a subset of the mouse lines, the β-galactosidase reporter gene (*LacZ*) was used to report about gene expression in 41 tissues, typically in heterozygotes of 6 weeks of age or older (4). The procedure includes the localization of the reporter gene in the adult mouse by observing the absence or presence of a staining in a particular tissue or organ. However, some of the tissues are considered difficult, e.g. as a background colour makes a confident call impossible. For this reason, we excluded the problematic tissues, leading to a subset of 26 non-gender-specific tissues and 5 gender-specific tissues. For each tissue, the expression value can be one of present (staining corresponding to the expression of the gene is clearly visible), absent (if no gene expression occurs in the investigated tissue), ambiguous (the annotator cannot take a clear decision from the image whether or not tissue expression is present) or no data (if no images have been taken). We included here only the ‘present’ calls and excluded all other calls. The data set comprised annotations for 383 genes and was downloaded from the Sanger-MGP BioMart on 4 November 2013.

The second expression data repository used here was the Gene Expression Barcode database. The Gene Expression Barcode database includes data from the public Gene Expression Omnibus and ArrayExpress repositories and harmonizes the results from MicroArray experiments into ‘absent/present’ calls across a range of human and mouse tissues using the method described in (18). We downloaded a snapshot of the mouse transcriptome data on 1 November 2012, and the download file comprised gene annotation data for 15 789 genes and 246 tissues.

A harmonized subset between both expression data files was formed by mapping the gene identifiers and limiting the tissues to the common subset. The reduced data set included 21 normal adult tissues, which were then manually aligned to Mouse Adult Gross Anatomy (MA) concepts (19) for data evaluation and integration purposes.

#### MGD marker information

The Gene Expression Barcode database provided their download file with MGD gene symbols and Ensembl identifiers (IDs) to reference mouse genes. We chose MGD gene accession IDs to be the point of reference. Therefore, we also downloaded the MGI_Gene_Model_Coord.rpt MGD report on 4 November 2013. The report file contained 38 236 Ensembl IDs together with their corresponding MGD gene accession IDs. Applying the report file data, we were able to map the expression data for 14 881 genes from the Gene Expression Barcode data. This reduced set of Gene Expression Barcode profiles was used in all subsequent analyses.

#### Ontology data

In a previous study, Mungall *et al.* (20) suggested the composition of species-specific phenotypes with species-agnostic ontologies to facilitate the comparison of phenotypes across species using ontologies and semantic similarity measures. This led to the generation of so-called entity-quality (EQ) statements by means of which a phenotype is decomposed into an affected entity (e.g. anatomical component or process) that is further described with a quality. For example, the phenotype micrognathia (MP:0002639) is composed using jaw (MA:0001905) as affected anatomical entity and decreased size (PATO:0000587) as descriptive quality. We downloaded the EQ statements for MP concepts from the Google project web page (**https://code.google.com/p/phenotype-ontologies/**) and extracted the MP concepts that contain at least one anatomical entity in their EQ statement. Anatomical entities are represented with UBERON concepts, a species-agnostic anatomy ontology (21). We then used UBERON’s cross-references to MA to associate MP concepts with MA concepts, leading to an association of 4000 MP concepts with 1162 unique MA concepts.

### Establishing connections between tissues and phenotypes

To establish connections between expression and phenotype data, Hoehndorf *et al.* (13) suggested the hypergeometric distribution for ‘absent/present’ calls in the expression data, in accordance with the extended FUNC software applied (22). A hypergeometric distribution provides the probability to draw a certain number of successes from a population without replacement. However, for small population sizes, as well as a small number of successes available in the population, the hypergeometric distribution may lead to a distorted view on the data and, consequently, false-positive associations between tissues and phenotypes. To provide more confidence in the obtained scores, we not only restricted the input parameters of the hypergeometric distributions (see *Hypergeometric distribution*) but also used a second method. We applied association rule mining as the second method, which has been previously used to successfully extract links between Gene Ontology [GO, (23)] concepts from the three different branches of GO (24–26). The links were extracted from large annotation sets.

#### Hypergeometric distribution

*P*-values calculated from hypergeometric distributions have been successfully applied in the bioinformatics domain. For example, a hypergeometric distribution was used to map chromosomal regions and vocabularies based on textual evidence (27) as well as finding enriched GO terms [or other ontologies (13)] for expressed genes (22). As described in (27), the *P*-value corresponds to the hypergeometric cumulative distribution function described by

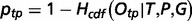
 with *O_tp_* the number of times tissue and phenotype are used in conjunction to annotate a gene; *T* being the number of times the tissue occurs as an annotation in the data set; *P* being the number of times the MP concept occurs as an annotation in the data set; *PT* being the overall count of genes and *p_tp_* being the resulting *P*-value.

Owing to the fact that a hypergeometric distribution may distort the view when the occurrences of MP concepts are low, we only included MP concepts that occur at least 10 times as annotations in the applied annotation set. After manual evaluation of a preliminary test run, we determined a *P*-value cut-off level of 0.005 (data not shown).

#### Association rule mining

Association rule mining was traditionally used to determine connections between items that are frequently purchased together, but it has been successfully applied in the bioinformatics domain to determine relationships between Gene Ontology concepts (24–26). Our goal was to determine rules existing between tissues and phenotypes based on an extensive annotation set, similar to the Gene Ontology studies. Therefore, we used association rule mining as our second approach to determine connections between tissues and phenotypes. We used here the *a priori* implementation (http://www.borgelt.net/doc/apriori/apriori.html) of association rule mining, setting the parameters to 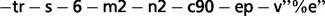

These parameters’ settings mean that only rules are extracted between the tissue and phenotype that possess a confidence level of at least 90%, co-occur at least six times together as annotations for a gene and the results are returned as *P*-values instead of probabilities.

### Implementation

While no additional implementation was required for the association rule mining, data preparation and harmonization as well as calculating the *P*-values using a hypergeometric distribution were implemented using Groovy, version 2.0.4 (http://groovy.codehaus.org/Download), and the Apache Commons Math library, version 3.3.2 (http://commons.apache.org/proper/commons-math/). To access the ontology files and extract *is_a* and *part_of* relations between MA concepts, we used Groovy in conjunction with the Brain library (version 1.4) (https://github.com/loopasam/Brain) (28), the Elk reasoner library (version 0.3.2) (https://code.google.com/p/elk-reasoner/) (29) and the OWL API library (version 3.2.3, as required by the Elk reasoner library) (http://owlapi.sourceforge.net/).

#### Implementation of web interface

PhenoDigm’s original web interface was developed using the Play! framework (http://www.playframework.org/) (version 1.2.5). The functionality of the Play! framework was extended using jQuery (version 1.6.4) and jQuery UI (version 1.9.1). As a consequence, the extension of the web interface that allows access to the association scores between tissues and phenotypes uses the same software technologies and versions.

### Evaluation

To evaluate the obtained tissue–phenotype associations, we conducted an automated as well as a manual evaluation. The details for both evaluation steps are provided in the following subsections.

#### Comparison with EQ statements of MP concepts

Using a compositional method as described by Mungall *et al.* (20), phenotypes can be described using anatomical entities that correspond to tissue types. To automatically evaluate our results, we used the EQ statements available for download for MP concepts. In total, 4000 MP concepts possessed an EQ statement, covering in total of 1162 MA concepts. Because the applied expression data covered only 21 tissues, we further filtered the EQ statements to those that can be represented with the tissues used in the study. Moreover, we allowed tissues that are either in a part_of or is_ a relationship (including transitivity) with the 21 tissues. This filtering step reduced the available evaluation set to 1546 MP concepts, covering 491 unique MA concepts.

In the automated evaluation step, we first assessed how many phenotypes obtain the expected tissue (allowing for subclass and ‘part_of’ relationships). Furthermore, we evaluate each individual phenotype–tissue association by means of whether this particular association of tissue and phenotype is expected. We note here that a tissue that is not found in the EQ statement may not necessarily be incorrect and may, in fact, constitute a biologically relevant case.

#### Manual investigations

Owing to the limitations of the automated evaluation, we also added a manual evaluation step to the workflow. We extracted those phenotypes from the results that did not obtain the expected tissue used in the EQ statement as any of the predicted tissue associations and investigated these cases further with respect to biological relevance as well as technical shortcomings of the method. This evaluation step was executed by two independently working curators, each evaluating a subset of the data. Results from both curators were merged to form the final evaluation results.

## Results and Discussion

To associate tissues and phenotypes based on gene expression and phenotype data, we used a hypergeometric distribution as well as the association rule mining. With the hypergeometric distribution, we obtained 2998 significant tissue associations for 1121 unique MP concepts. Applying the *a priori* software for association rule learning, we obtained 272 associations, including 205 unique MP concepts. The combined results from both methods constitute 3168 phenotype–tissue associations, comprising 1239 phenotypes. We evaluated these associations both automatically and manually.

### Comparison with logical definitions

First, we automatically evaluated the results using the EQ statements for MP concepts (see *Comparison with EQ statements of MP concepts*), assuming that the tissue used for the composition of a phenotype would also be one of the predicted tissues using either method. We allowed for the tissue to be counted as a match, if either the tissue predicted or the tissue used in the EQ statement is a part or subclass of the other. Results were evaluated on a phenotype level by means of whether the tissue from the EQ statement is contained in the predictions, but additional tissues may also be associated. All results are summarized in Table 1.

**Table 1.** Obtained evaluation results for the automated evaluation against tissues contained in EQ statements of MP concepts

	Hypergeometric distribution		Association rule	
Type of comparison	Phenotype	Association	Phenotype	Association
Expected - exact	184 (76%)	59	20 (71%)	2
Expected - psp		88		6
Expected - ldsp		66		13
Number of tissue matches	58 (24%)	373	8 (29%)	26
Number of EQ	879	2412	177	225
Total	1121	2998	205	272

Results are grouped by the type of comparison either on a phenotype or an individual phenotype-tissue association level. On the association level, expected tissues are further divided into whether the same tissue was predicted as used in EQ (exact), predicted tissue is subclass or part_of tissue in EQ (psp), or tissue in EQ is subclass or part_of tissue predicted (ldsp). Note that even though tissues between EQ and prediction do not match, the association may still be correct. No EQ means that no EQ statement referring a tissue that is related to any of the 21 applied was available for evaluation.

#### Comparison of hypergeometric distribution associations

Using the hypergeometric distribution led to the extraction of 2998 associations between a tissue and a phenotype, covering 1121 unique phenotypes. From the 1121 unique phenotypes, 242 possessed an EQ statement, whereas 879 either did not possess an EQ statement or the tissue was not represented with the 21 tissues used in this study (see *Comparison with EQ statements of MP concepts*). For 184 phenotypes out of these 242 (76%), we were able to recover the tissue used in the EQ statement. However, more than one tissue can be assigned to any of the 184 phenotypes. The remaining prediction results for 58 phenotypes (24%) did not include the tissue used in the logical definition.

For automated evaluation, 586 individual phenotype–tissue associations were available, as the phenotype had a suitable EQ statement. Of the 586 associations, 59 (10%) constituted exact matches by means that the tissue predicted is also used in the EQ statement of the phenotype. For 88 associations (15%), the predicted tissue is either a ‘part_of’ or subclass of the tissue used in the EQ statement. In 66 cases (11%), the tissue used in the EQ statement is a ‘part_of’ or a subclass of the tissue provided by the predicted associations. This leaves a total of 373 associations (64%), where the predicted tissue cannot be confirmed using the EQ statement. We note here that although the 373 associations cannot be confirmed using the EQ statements, it does not mean that they are incorrect biologically because the site of expression and the eventual manifestation of the phenotype do not have to directly correspond.

Our results show that for at least 76% of the phenotypes that could automatically be evaluated, the expected tissue is assigned. This suggests that the hypergeometric distribution can be applied to determine associations between tissues and phenotypes. Assuming that the tissue used in the EQ statement is correct, the 373 associations, where a different tissue was found to be associated with the phenotype, could be indicators for a spatial separation of gene expression and the resulting phenotype. These cases require further investigation and could potentially give novel biological insights.

#### Comparison of association rule mining results

By applying the association rule mining to the data set to identify connections between tissues and phenotypes, 272 rules including a tissue and a phenotype were generated. These 272 association rules, covering 205 unique phenotypes, were also automatically evaluated against the EQ statements of MP concepts (se*e Comparison with EQ statements of MP concepts*). From the 205 unique phenotypes, 177 either did not possess an EQ statement or the tissue used in the EQ statement could not be represented with the tissues used in this study. From the remaining 28 phenotypes, 20 (72%) obtained the expected tissue as one of the predictions, whereas 8 (28%) were not associated with the tissue used in the EQ statement.

For automated evaluation, 44 individual phenotype–tissue associations were available, as the phenotype had a suitable EQ statement. Two (4%) showed an exact match to the predicted tissues; in six cases (13%), the prediction was either a subclass or a ‘part_of’ the tissue used in the EQ statement; in 13 cases (28%), the tissue used in the EQ statement was a ‘part_of’ or subclass of the predicted tissue; and in 26 association rules (55%), the tissue predicted was not in a relationship with the tissue used for the EQ statement. Again, as discussed previously, these non-aligning associations are not necessarily wrong and are worthy of further biological investigations.

Using association rule mining on the applied annotation set provides a smaller set of associations between tissues and phenotypes compared with the hypergeometric distribution. However, the results of the automated evaluation suggest a similar behaviour. In total, 72% of the phenotypes obtain a predicted association with the tissue used in the EQ statements, while the remaining 28% need not be wrong associations. Our results show that association rule mining is, compared to the hypergeometric distribution, equally applicable to the task of learning associations between tissues and phenotypes even though the result set is comparatively smaller. The parameters applied for the *a priori* software may be the reason for the small result set, and future refinement may lead to more associations.

### Manual evaluation results

To further assess the quality of our results, we manually evaluated the phenotypes that did not obtain the expected tissue used in the EQ statements as a predicted association from the applied method, i.e. 8 phenotypes from the association rule mining and 58 phenotypes resulting from associations made with the hypergeometric distribution.

In the case of the association rule results, five of the manually investigated phenotypes only possess rules with fairly high *P*-values (in the range from 0.0036 to 0.015). This suggests that the applied parameters for the software may not be ideal, and a simple filtering of the rules according to the *P*-value may eliminate all tissue associations for these phenotypes. However, three of eight phenotypes show low *P*-values with at least one tissue: ‘abnormal ciliary body morphology’ (MP:0005099) with ‘skin’ (MA:0000151), *P* = 1.3e-06; ‘decreased spleen iron level’ (MP:0008808) with ‘liver’ (MA:0000358), *P* = 7.8e-04 and ‘eye opacity’ (MP:0009859) with ‘brain’ (MA:0000168), *P* = 1.7e-04.

For the ‘abnormal ciliary body morphology’ and ‘eye opacity’ examples, many of the supporting genes are also expressed in the expected tissue of the eye, but the data set did not allow for this connection to be made with association rule mining. We note here that this association can, however, be seen when using the hypergeometric distribution. Thus, it is possible that the phenotype is associated with expression in the expected tissue and is simply missed because of the stringency levels used in the association rule mining approach. In the example of ‘abnormal ciliary body morphology’*,* like ‘skin’, the ciliary body contains melanocytes, and melanomas of the ciliary body are relatively common (30). It is possible that the genes associated with ‘abnormal ciliary body morphology’ are expressed in melanocytes.

In the case of the ‘decreased spleen iron level’ and ‘liver’ association, there is no evidence that the supporting genes are expressed in the spleen as well. The hepatic peptide hepcidin is known to control the amount of iron stored in the bone marrow, liver and spleen (31). Thus, it is possible that genes expressed in the ‘liver’ and associated with ‘decreased spleen iron level’ are involved in the regulation of hepcidin. The *Hamp* gene that expresses hepcidin is one of the genes contributing to our association.

In the case of the hypergeometric distribution, 26 of the 59 evaluated phenotypes had the expected tissue assigned but the *P-*values were in the range of 0.005–0.5. To avoid a high number of misleading annotations, we set the cut-off *P*-value to 0.005. While this means that some of the expected tissues (with respect to the tissue used in the EQ statement) may disappear, overall, only highly significant connections between tissue and phenotypes will be reported that likely signal a relevant biological connection. We note here that the predictions are only as good as the annotation set. However, as in the case of the association rule mining, a number of potentially relevant biological connections could be identified based on the obtained *P*-values: ‘decreased total body fat amount’ (MP:0010025) and ‘brain’ (MA:0000168), *P* = 6.3e-7; ‘liver hypoplasia’ (MP:0000600) and ‘spleen’ (MA:0000141), *P* = 5.4e-6; and ‘lung inflammation’ (MP:0001861) and spleen (MA:0000141), *P* = 9.9e-5.

The ‘liver’ and ‘spleen’ work together closely in the maintenance of red blood cells and co-occurring abnormalities are often seen, e.g. hepatosplenomegaly. Hence, it is plausible that disruptions in spleen-expressed genes could affect the liver, resulting in ‘liver hypoplasia’. The *spleen* also plays a vital role in the immune system, so the ‘lung inflammation’ phenotype associated with disruption of spleen-expressed genes could be a consequence of the de-regulation of an immune response.

The final example of an association between genes expressed in the ‘brain’ and ‘total body fat’ abnormalities is intriguing because, as described in the Introduction section, many recent studies have highlighted the role that genes expressed in the ‘brain’ play a role in obesity. The list of genes associated with ‘body fat abnormalities’ and brain expression in the mouse may be interesting candidates to consider in future obesity studies.

### Application of data to use cases

The original PhenoDigm application was designed to prioritize candidate genes for diseases by semantically comparing the clinical features with the phenotypes of mouse mutants involving the ortholog of the gene. Incorporating expression data as well as phenotype comparisons has the potential to reduce or re-prioritize the set of candidates.

For example, *Familial Erythrocytosis 1* (OMIM:133100) is known to result from mutations in the *JAK2* gene. PhenoDigm reveals that mouse models disrupting the mouse ortholog exhibit highly similar phenotypic features to the disease, but there are models involving six other genes that score higher (www.sanger.ac.uk/resources/databases/phenodigm/disease/OMIM:133100). If we take the best MP matches (e.g. *enlarged spleen* and *thrombophlebitis*) for the clinical phenotypes associated with this disease, five tissues are associated with these MP terms in our association set involving the spleen, liver, lung, white adipose tissue and mammary gland. Restricting the top 200 PhenoDigm candidates to only genes expressed in these five tissues reduces the set to 26 genes, and *JAK2* is now the top candidate.

### Browsing data online

**Figure 2.**
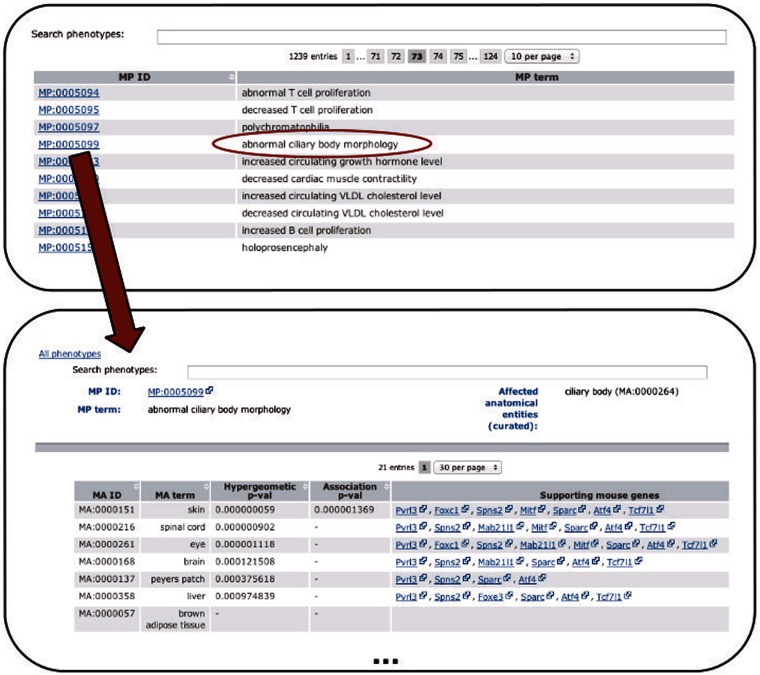
Depicts the extension of the PhenoDigm web interface and how the data can be browsed using the newly added pages. From a list of phenotypes, those of interest can be selected, leading to a page that shows *P*-values for each of the investigated tissues. Hyphens in one of the *P*-value fields indicate that with the data set no significant association between the phenotype and the tissue could be identified. For significant associations, genes supporting the association between tissue and phenotype are provided.

After evaluating the obtained associations, we included the significant tissue–phenotype connections into PhenoDigm’s original web interface. The data can be browsed starting from the phenotype level. Once a phenotype is chosen, all significant tissues are provided together with the EQ statement, where an EQ statement is available. *P*-values from either method are kept and provided to the user of the online data. For each significant connection, all genes that are annotated with both the MA as well as the MP concept are provided. These genes can then be further investigated using the gene pages available from MGD or any other tool. An illustration of the workflow and structure of the data is available in Figure 2.

While the data are, at the moment, an extension to the PhenoDigm’s original database, we intend to link both data sets further together in future works and provide an integrated view of the data.

## Conclusions and Future Work

In conclusion, we established potentially biologically relevant associations between tissues and phenotypes and evaluated the obtained results using an automated as well as a manual evaluation step. We obtained a total of 3168 significant associations (covering 1239 phenotypes) that are provided as an extension to PhenoDigm’s web interface and can be browsed online. These associations can be applied in further use cases, e.g. to narrow down the disease gene candidates or drug targets predicted from other methods.

In future works, we intend to further assess the importance of *P*-value thresholds and the ideal parameter settings for the *a priori* software applied. In addition, we plan to incorporate expression data from developmental stages and not only from adult tissues. Some of the mouse lines analyzed are embryonic lethal or subviable, and, in addition, some of the phenotypes recorded for adult lines will have initially arisen during development. Incorporation of expression data from embryonic stages as well may allow for the extraction of additional associations to these developmental phenotypes.

We also plan to incorporate more tissues than the 21 included here. The Gene Expression Barcode was (see *Expression data*), for example, significantly reduced when establishing the common subset with Sanger MGP expression data. An ideal number of tissues needs to be determined to still obtain significant associations between tissues and phenotypes. After the intended improvements, the data will be updated in the web interface and further linked with the original data contained in PhenoDigm.

## Author Contributions

A.OE designed the study, implemented the Groovy scripts required for the analysis of the data and partially executed the manual evaluation of the data. D.S assisted with evaluation, implemented the extension to PhenoDigm’s web interface and facilitated the data import into PhenoDigm's database. Both contributed to the final version of this manuscript.

## Funding

This work was supported by the Wellcome Trust grant [098051] and the National Institutes of Health (NIH) grant [1 U54 HG006370-01]. Funding for open access charge: Wellcome Trust grant [098051] and the National Institutes of Health (NIH) grant [1 U54 HG006370-01].

*Conflict of interest*. None declared.
